# Supplementary material for: Hypoxia-induced inhibin promotes tumor growth and vascular permeability in ovarian cancers
Source: Commun Biol. 2022 Jun 2;5:536. doi: 10.1038/s42003-022-03495-6 (PMC9163327; doi:10.1038/s42003-022-03495-6)
Supplement: Supplementary file 2 — Supplementary Information [file 42003_2022_3495_MOESM2_ESM.pdf]

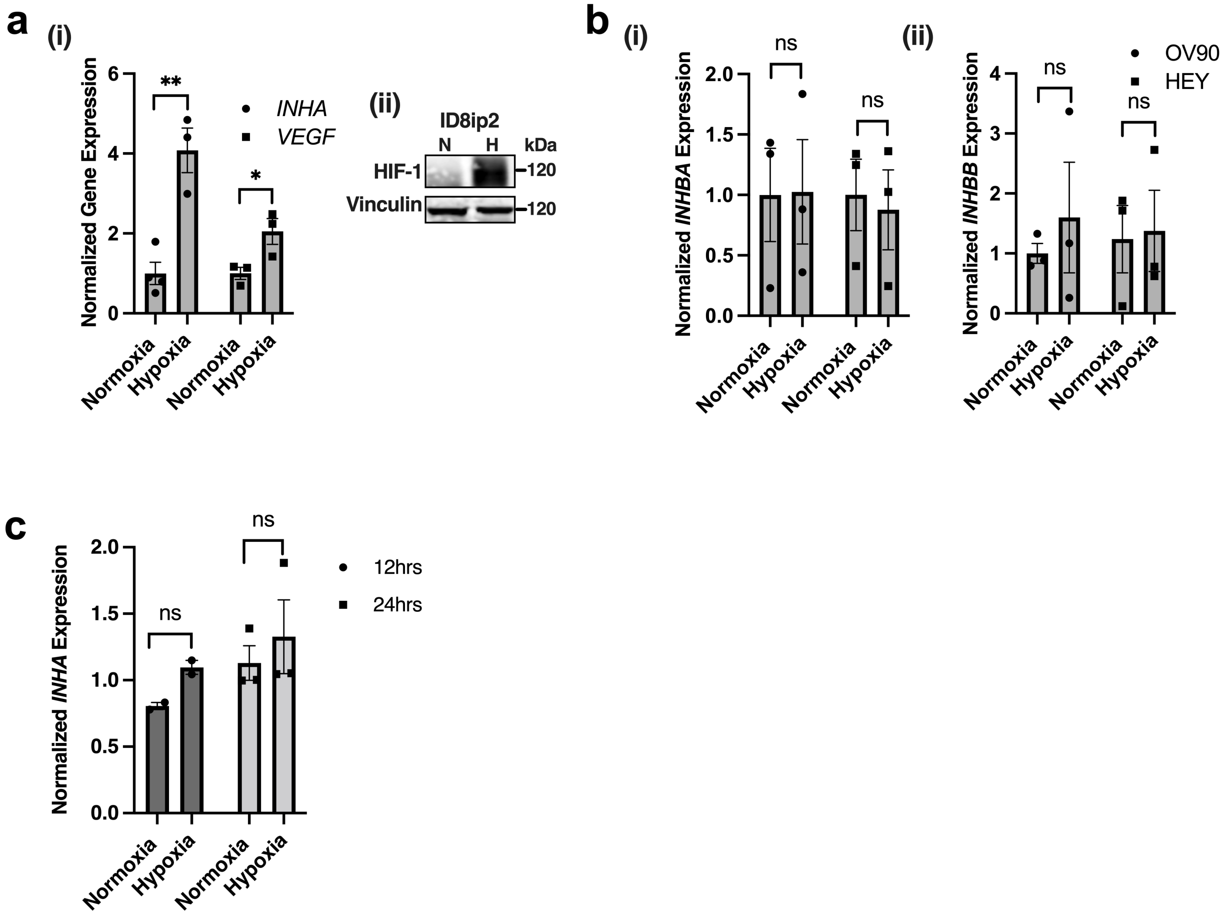

Supplementary Figure 1. a) Relative qRT-PCR analysis of *INHA* and *VEGFA* mRNA expression normalized to corresponding levels in normoxia in ID8ip2 cells grown under hypoxia (0.2%) or normoxia (17-21%) for 24hrs. Mean±SEM, n=3. *, p<.05; **, p<.01, unpaired t-test. b) Relative qRT-PCR analysis of (i) *INHBA* and (ii) *INHBB* mRNA expression normalized to corresponding levels in normoxia in OV90 or HEY cells grown under hypoxia (0.2%) or normoxia (17-21%) for 24hrs. Mean±SEM, n=3. n.s., not significant, unpaired t-test. c) Relative qRT-PCR analysis of *INHA* mRNA expression normalized to corresponding levels in normoxia in HMEC-1 cells grown under hypoxia (0.2%) or normoxia (17-21%) for either 12 or 24hrs. Mean±SEM, n=3 for 12hrs and 2 for 24hrs. n.s., not significant, unpaired t-test.

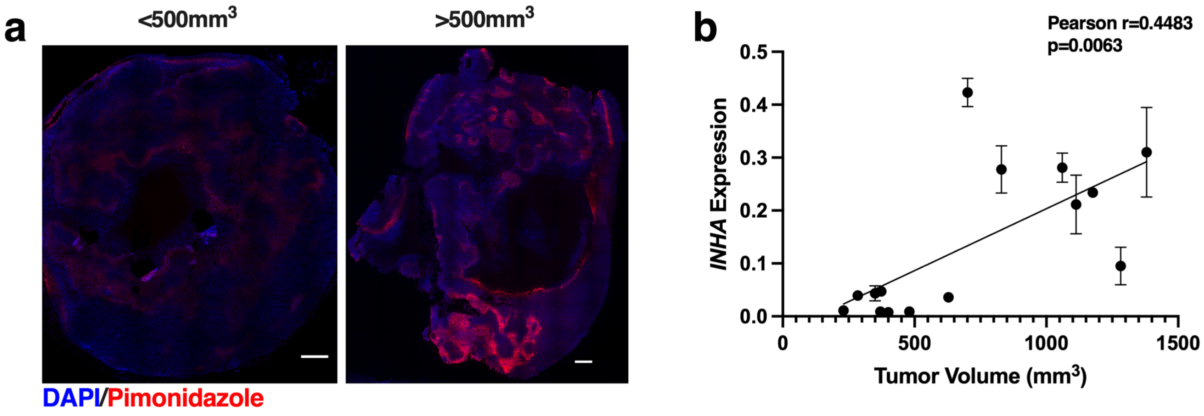

Supplementary Figure 2. a**)** Representative images of pimonidazole staining in HEY xenograft tumors used for calculation of hypoxic area. Scale bar: 500μm. b) Correlation analysis of *INHA* expression and HEY subcutaneous tumor volume (mm^3).

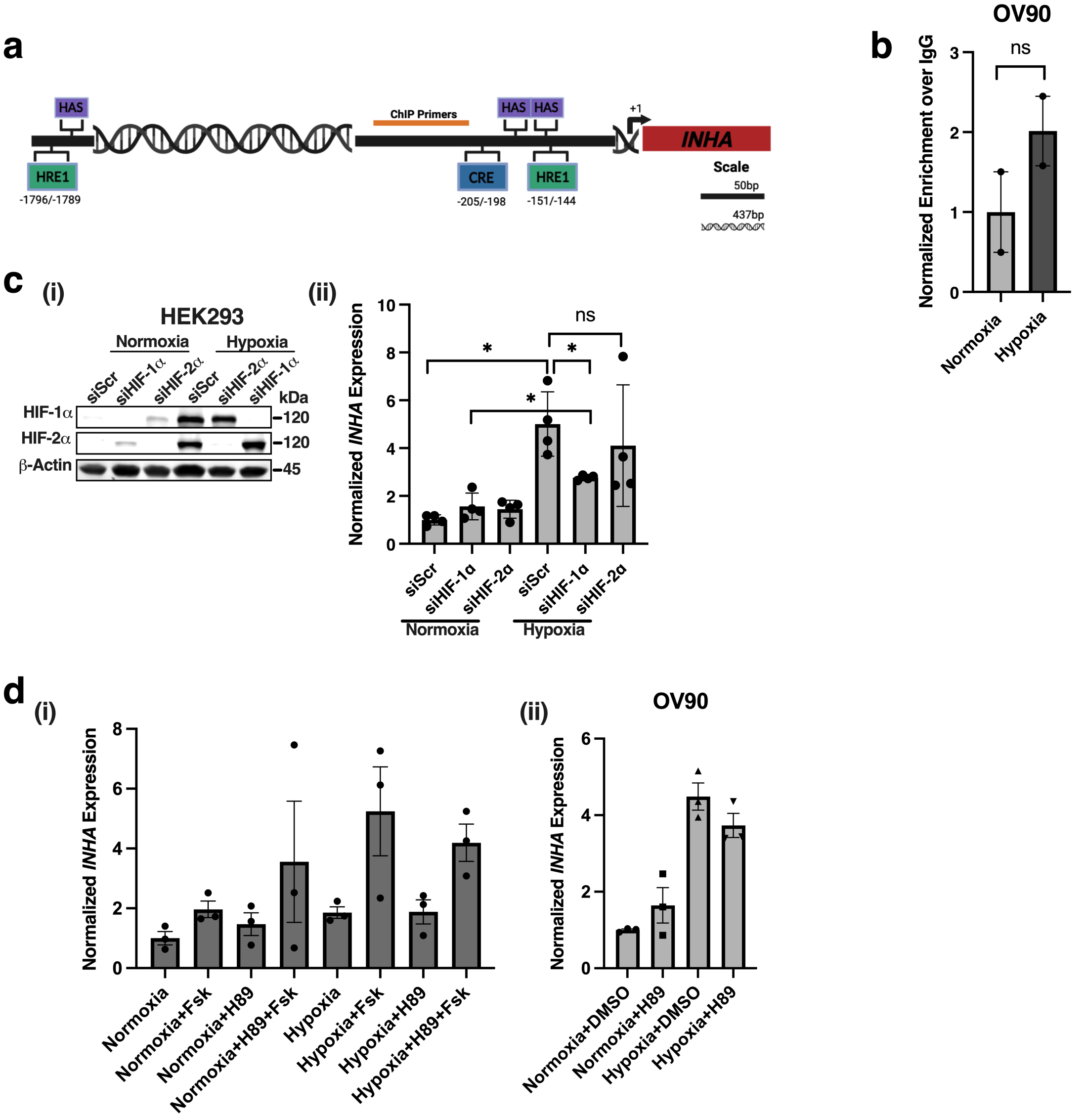

Supplementary Figure 3. a) Schematic of *INHA* promoter containing location of hypoxia response elements (HRE), hypoxia ancillary sequences (HAS), cAMP response element (CREB), and ChIP primers. Schematic is drawn to scale with appropriate scale information below. b) Relative qRT-PCR analysis using primers that amplify the distal HRE region in Supplementary Fig. 3A after chromatin immunoprecipitation (ChIP) of HIF-1 with HIF-1α antibody in OV90 cells. ChIP qRT-PCR results were quantified as normalized enrichment over IgG and normalized to normoxia. Mean±SEM, n=2. n.s., not significant. c) (i) Representative western blot and (ii) relative qRT-PCR analysis of *INHA* expression from HEK293 cells transfected with either siScr, siHIF-1α or siHIF-2α and exposed to hypoxia (0.2% O_2) for 24hrs. Mean±SEM, (n=4). *, p<.05; ***, p<.001; ****, p<.0001, One-way ANOVA followed by Tukeys multiple comparison test d) (i) Relative qRT-PCR analysis of *INHA* mRNA from ID8ip2 cells treated with either 10µM H89 or 20µM forskolin (Fsk), grown under normoxia or hypoxia 0.2% O_2 for 24hrs and normalized to normoxia. Mean±SEM, n=3. (ii) Relative qRT-PCR of *INHA* mRNA from OV90 cells treated with 10µM H89 and grown under normoxia or hypoxia (0.2% O_2) for 24hrs and normalized to normoxia. Mean±SEM, n=3.

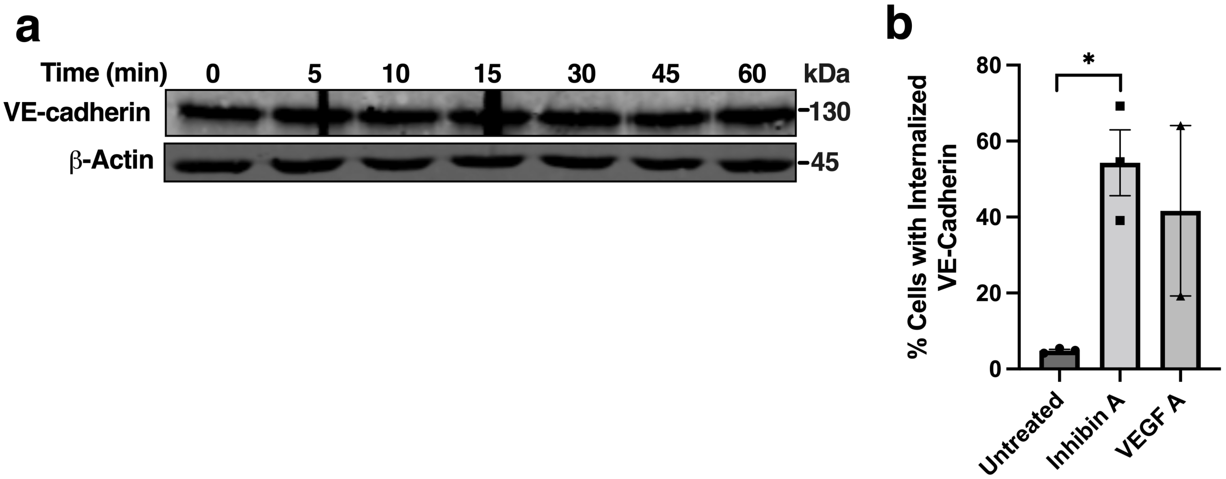

Supplementary Figure 4. a) HMEC-1 grown to confluence were treated with 1nM Inhibin A for indicated time. Lysates were immunoblotted for VE-cadherin and β-Actin. b) Percentage of HMEC-1 with internalized VE-cadherin after treatment with either 1nm inhibin A or VEGF A. *, p<.05, unpaired t-test.

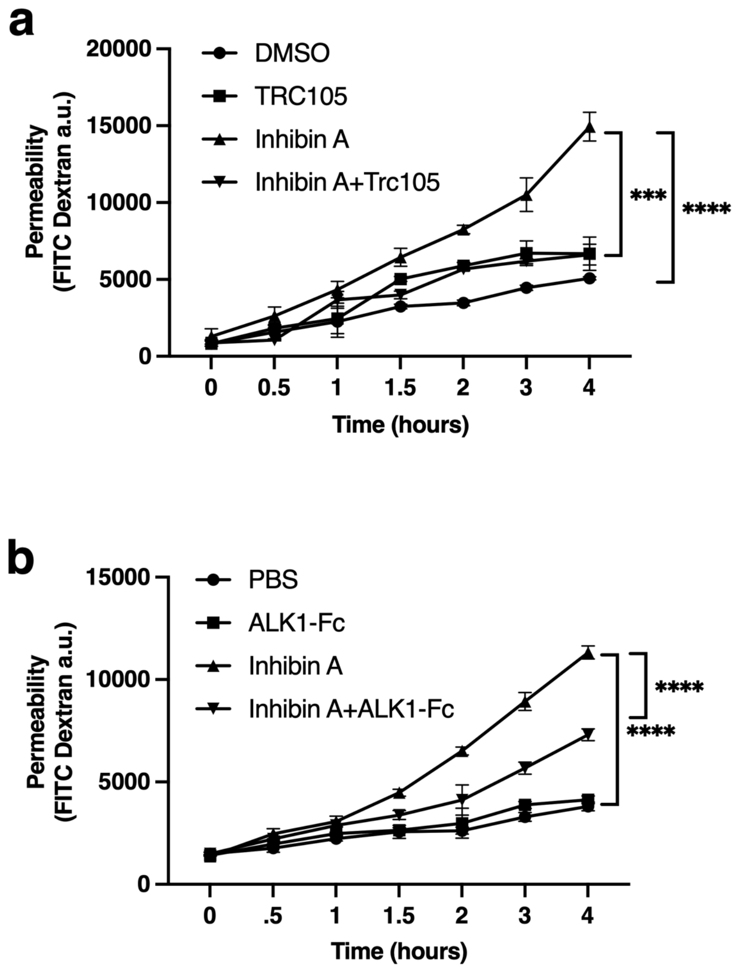

Supplementary Figure 5. Endothelial cell permeability of a HMEC-1 monolayer grown on matri-gel coated 3μm trans-well filters and treated with 1nM inhibin A in the presence or absence of either a) 100μg/mL TRC-105 or b) 10ng/mL ALK1-Fc. FITC-dextran diffusion was measured across the HMEC-1 monolayer at indicated times. Mean±SEM, n=4 for A and n=3 for B. for all conditions except TRC105+ML347 (n=3). ***, p<.001; ****, p<.0001.

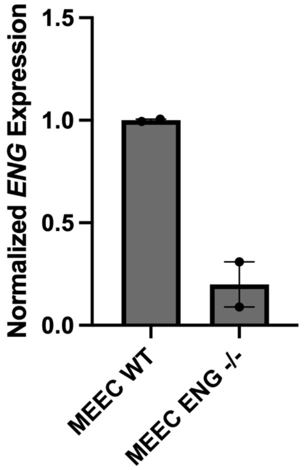

Supplementary Figure 6. Relative qRT-PCR analysis of ENG mRNA expression in MEEC WT or MEEC ENG-/-.

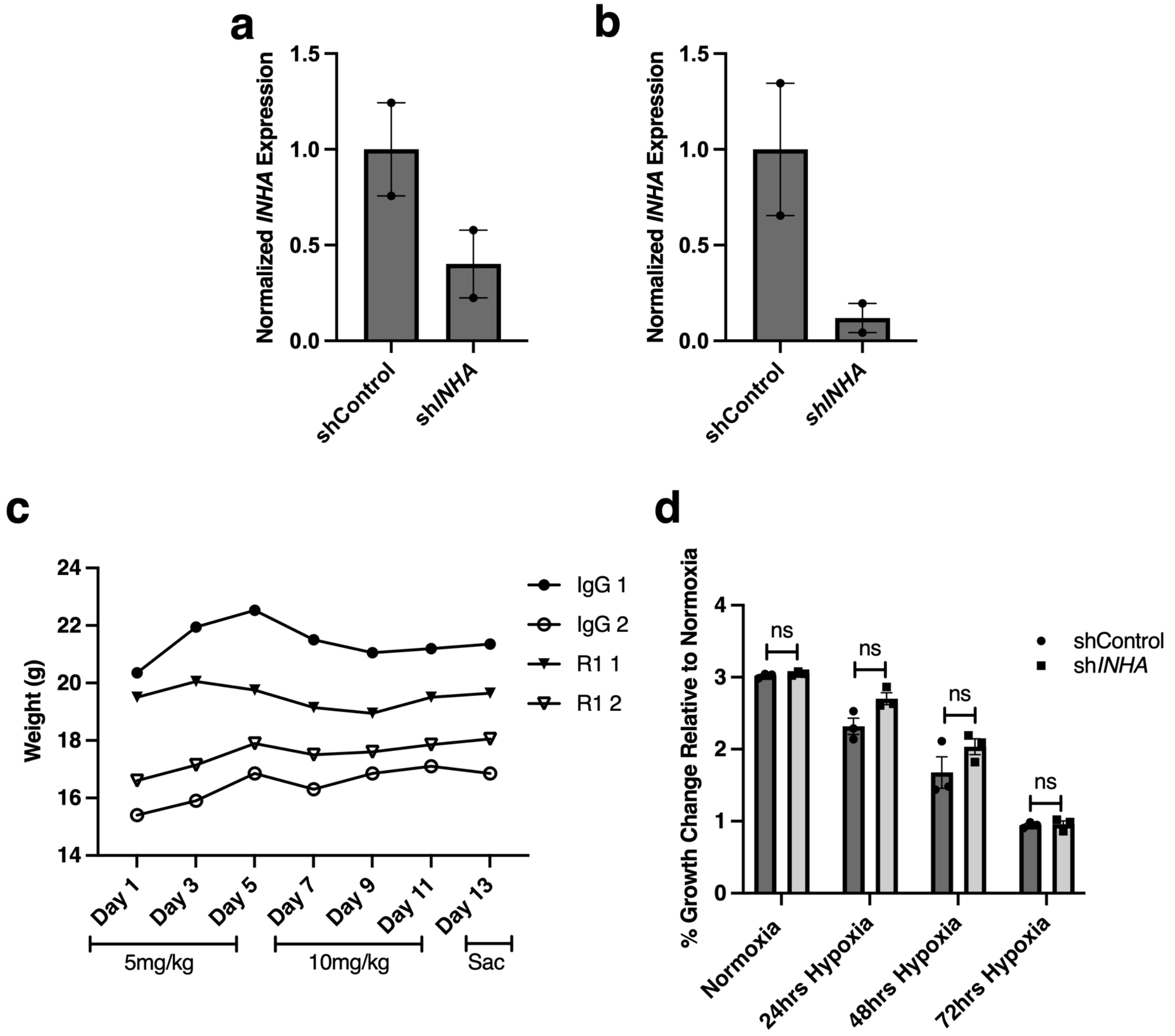

Supplementary Figure 7. a-b) Relative qRT-PCR analysis of *INHA* mRNA expression in HEY shControl and sh*INHA* cells (a) prior to subcutaneous injection and (b) at end point after harvest normalized to shControl. Mean±SEM, n=2. c) Weight of Ncr nude mice across two-week time course of anti-inhibin R1) or IgG antibody delivery. Injections were given every other day starting at 5mg/kg and escalated to 10mg/kg after 7 days. d) Growth curves of HEY shControl or sh*INHA* after indicated time under hypoxia (0.2% O_2). Data is normalized to growth under normoxia after 72hrs. Mean±SEM, n=2. n.s., not significant, unpaired t-test.

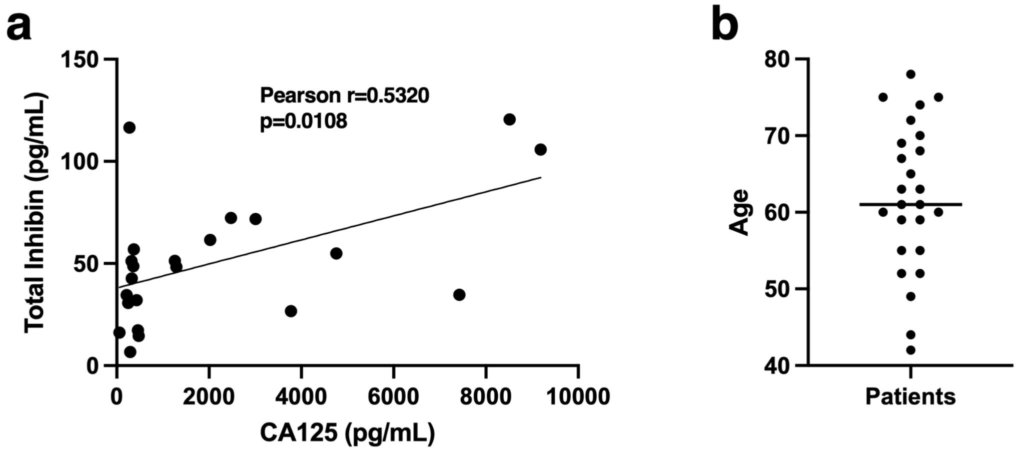

Supplementary Figure 8. a) Correlation analysis of total inhibin (pg/mL) and CA125 (pg/mL) in patient ascites. b) Median age of patients used for patient ascites ELISA analysis.

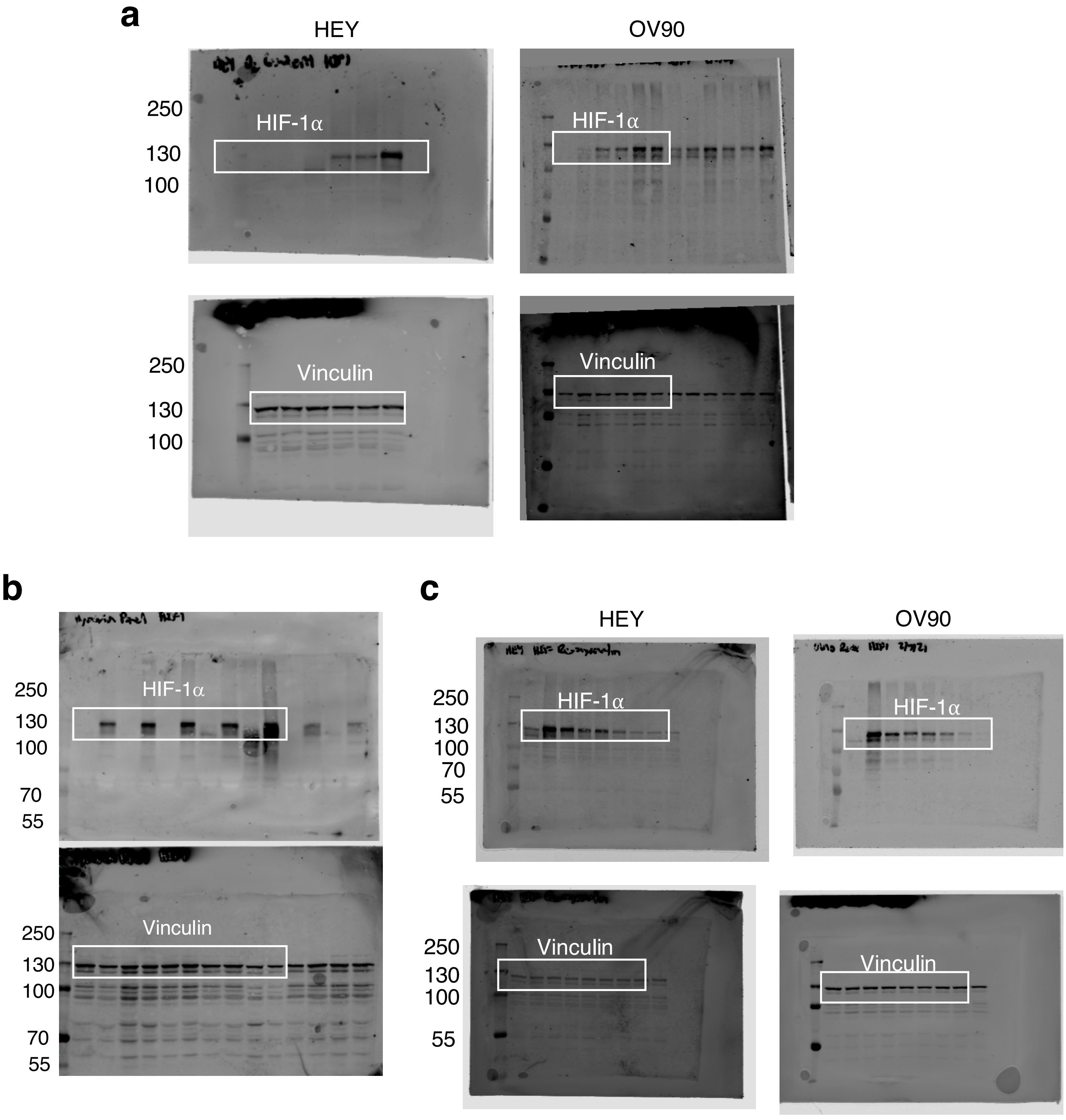

Supplementary Figure 9. Uncropped blots from a) Fig. 1a, b) Fig. 1b and Supplementary Fig. 1a, and c) Fig. 1d.

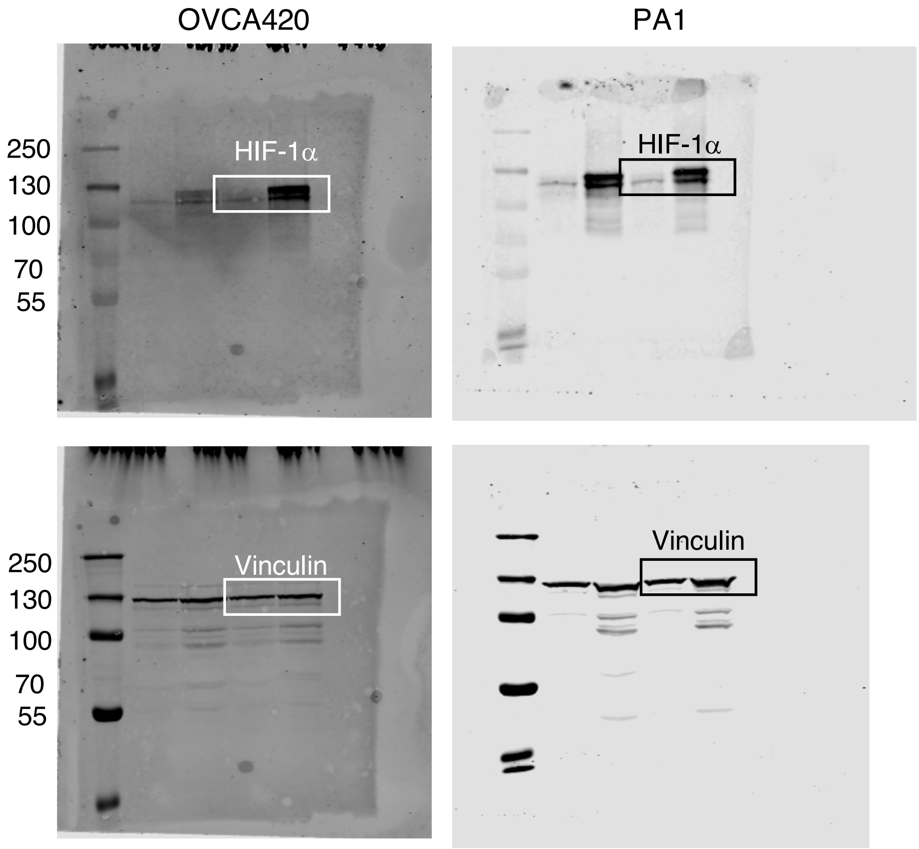

Supplementary Figure 10. Uncropped blots from Fig. 2a.

a

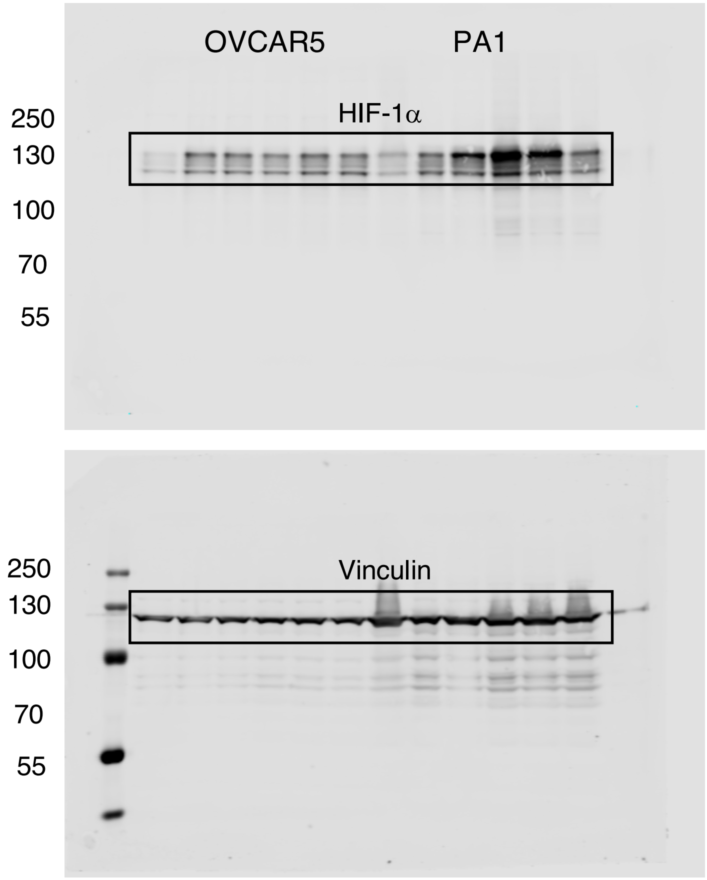

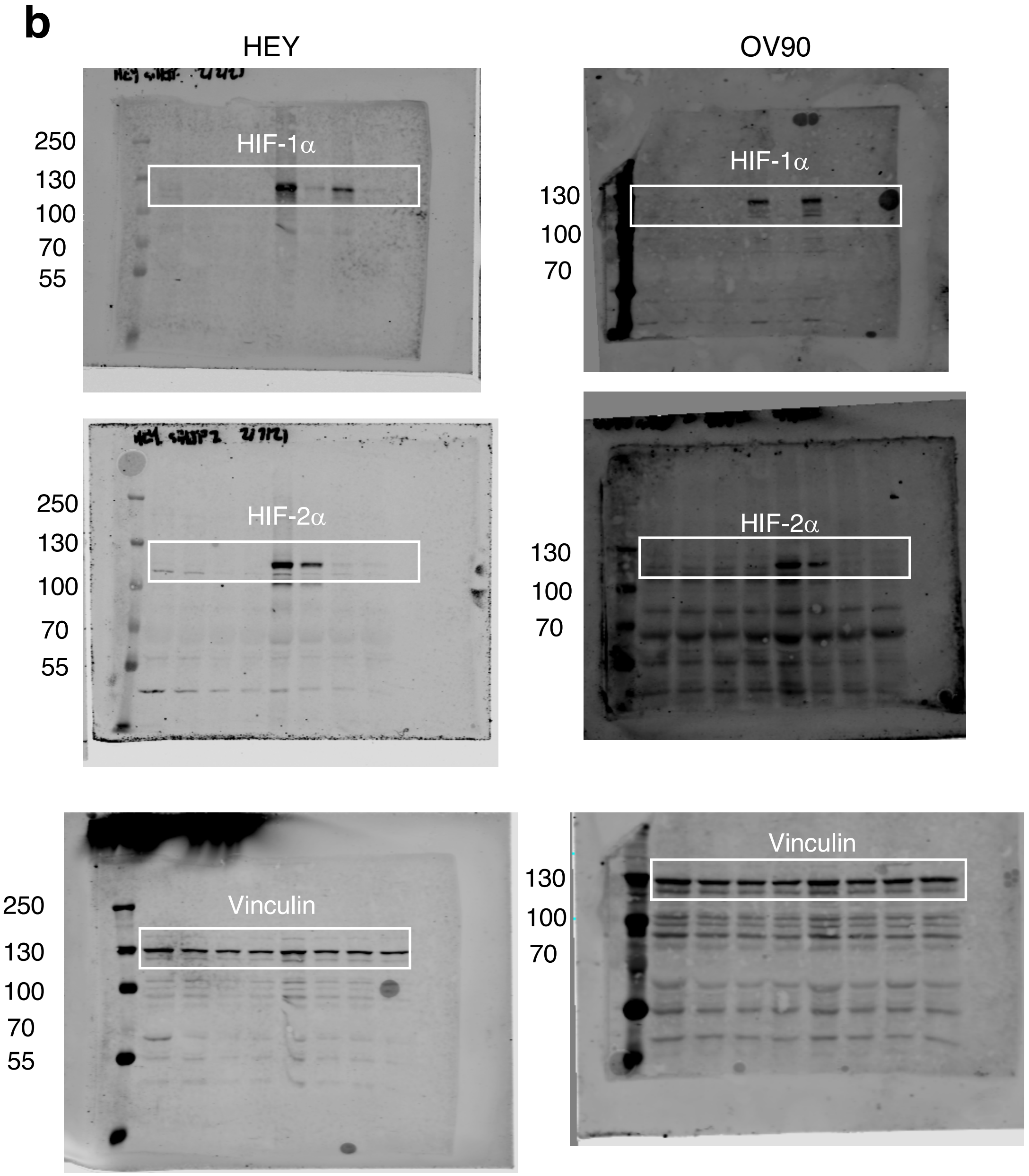

Supplementary Figure 11. Uncropped blots from a) Fig. 3a and b) Fig. 3c.

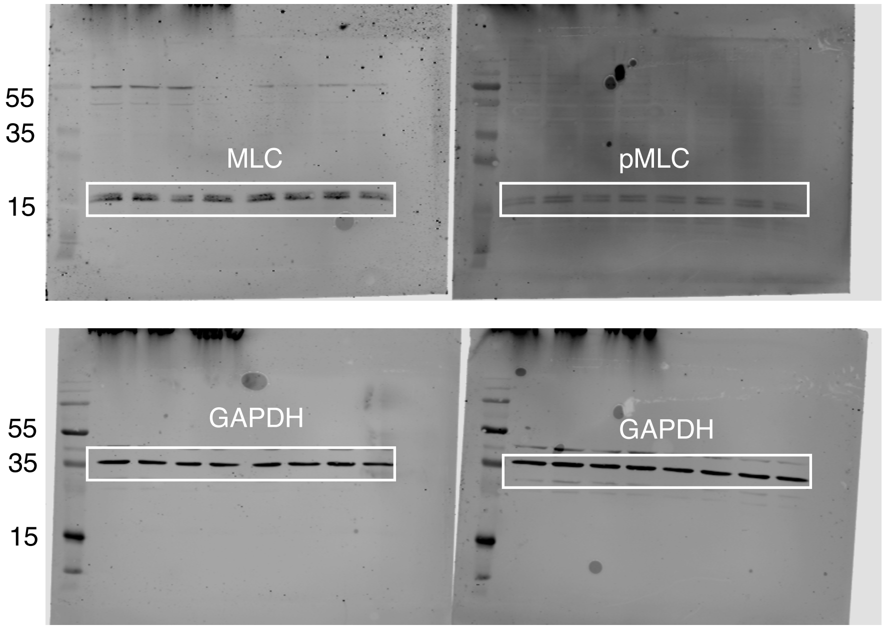

Supplementary Figure 12. Uncropped blots from Fig. 5b.

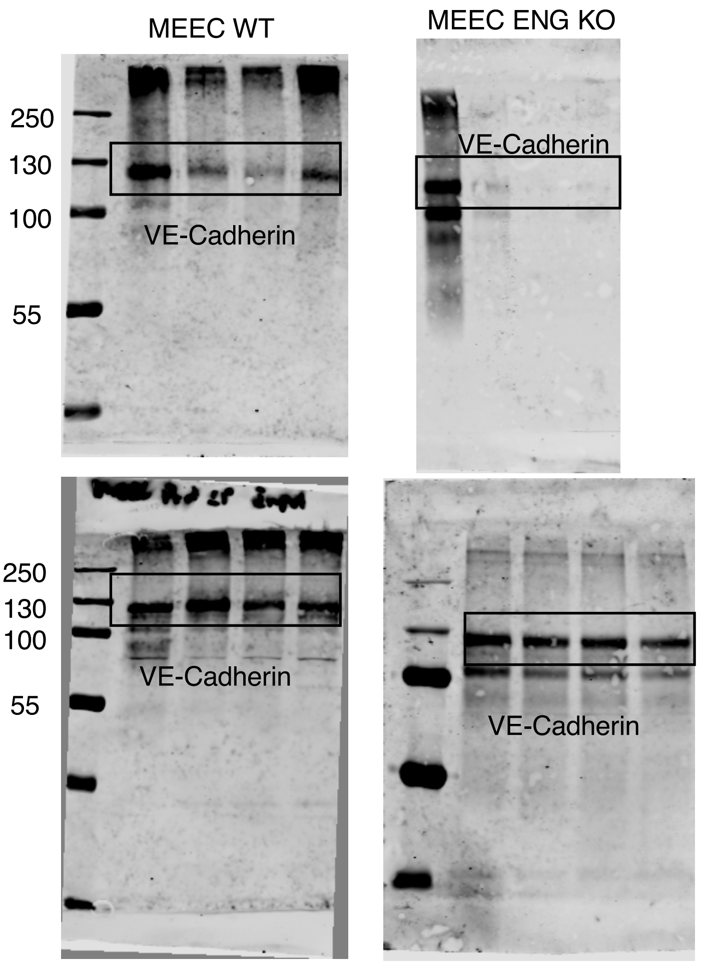

Supplementary Figure 13. Uncropped blots from Fig. 6b.

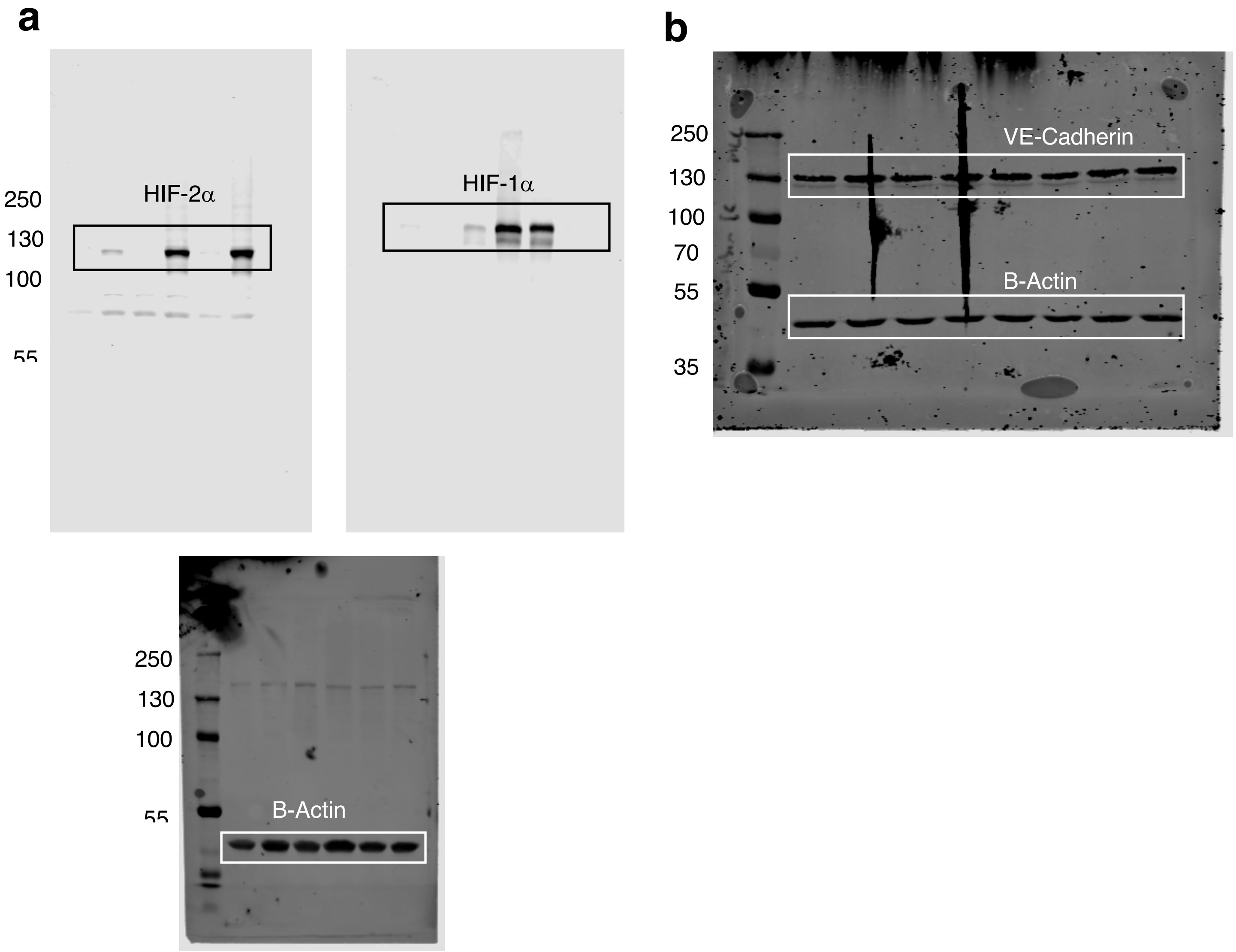

Supplementary Figure 14. Uncropped blots from a) Supplementary Fig. 3c and b) Supplementary Fig. 4a.
