# Supplementary material for: Hypoxia-induced inhibin promotes tumor growth and vascular permeability in ovarian cancers
Source: Commun Biol. 2022 Jun 2;5:536. doi: 10.1038/s42003-022-03495-6 (PMC9163327; doi:10.1038/s42003-022-03495-6)
Supplement: Supplementary file 3 — Description of Additional Supplementary Files [file 42003_2022_3495_MOESM3_ESM.pdf]

Description of Additional Supplementary Files

File name: Supplementary Data 1

Description: Underlying source data for all graphs and charts.

File name: Supplementary Data 2

Description: Key Resources Table
