# Supplementary material for: Hypoxia-induced inhibin promotes tumor growth and vascular permeability in ovarian cancers
Source: Commun Biol. 2022 Jun 2;5:536. doi: 10.1038/s42003-022-03495-6 (PMC9163327; doi:10.1038/s42003-022-03495-6)
Supplement: Supplementary file 6 — Reporting Summary [file 42003_2022_3495_MOESM6_ESM.pdf]

nature portfolio

Corresponding author(s): Karthikeyan, Mythreye

Last updated by author(s): Apr 5, 2022

Reporting Summary

Nature Portfolio wishes to improve the reproducibility of the work that we publish. This form provides structure for consistency and transparency in reporting. For further information on Nature Portfolio policies, see our Editorial Policies and the Editorial Policy Checklist.

Statistics

For all statistical analyses, confirm that the following items are present in the figure legend, table legend, main text, or Methods section.

n/a	Confirmed	
☐	☒	The exact sample size (n) for each experimental group/condition, given as a discrete number and unit of measurement
☐	☒	A statement on whether measurements were taken from distinct samples or whether the same sample was measured repeatedly
☐	☒	The statistical test(s) used AND whether they are one- or two-sided<br>*Only common tests should be described solely by name; describe more complex techniques in the Methods section.*
☒	☐	A description of all covariates tested
☐	☒	A description of any assumptions or corrections, such as tests of normality and adjustment for multiple comparisons
☒	☐	A full description of the statistical parameters including central tendency (e.g. means) or other basic estimates (e.g. regression coefficient) AND variation (e.g. standard deviation) or associated estimates of uncertainty (e.g. confidence intervals)
☐	☒	For null hypothesis testing, the test statistic (e.g. F, t, r) with confidence intervals, effect sizes, degrees of freedom and P value noted<br>*Give P values as exact values whenever suitable.*
☒	☐	For Bayesian analysis, information on the choice of priors and Markov chain Monte Carlo settings
☒	☐	For hierarchical and complex designs, identification of the appropriate level for tests and full reporting of outcomes
☐	☒	Estimates of effect sizes (e.g. Cohen's d, Pearson's r), indicating how they were calculated

Our web collection on statistics for biologists contains articles on many of the points above.

Software and code

Policy information about availability of computer code

Data collection	No software was used
Data analysis	Prism 9 was used for statistical analysis.

For manuscripts utilizing custom algorithms or software that are central to the research but not yet described in published literature, software must be made available to editors and reviewers. We strongly encourage code deposition in a community repository (e.g. GitHub). See the Nature Portfolio guidelines for submitting code & software for further information.

Data

Policy information about availability of data

All manuscripts must include a data availability statement. This statement should provide the following information, where applicable:

- Accession codes, unique identifiers, or web links for publicly available datasets
- A description of any restrictions on data availability
- For clinical datasets or third party data, please ensure that the statement adheres to our policy

All data generated or analyzed during this study are included in this published article and its supplementary information files.

March 2021

Field-specific reporting

Please select the one below that is the best fit for your research. If you are not sure, read the appropriate sections before making your selection.

[x] Life sciences [ ] Behavioural & social sciences [ ] Ecological, evolutionary & environmental sciences

For a reference copy of the document with all sections, see nature.com/documents/nr-reporting-summary-flat.pdf

Life sciences study design

All studies must disclose on these points even when the disclosure is negative.

Sample size
Sample size for animal studies was determined based on pilot experiments.

Data exclusions
No data was excluded from the analysis.

Replication
Yes, attempts at replication were successful.

Randomization
Allocation was random as animals were randomly distributed after receipt from commercial vendor

Blinding
Investigators were blinded to group allocation but were not blinded to data collection or analysis.

Reporting for specific materials, systems and methods

We require information from authors about some types of materials, experimental systems and methods used in many studies. Here, indicate whether each material, system or method listed is relevant to your study. If you are not sure if a list item applies to your research, read the appropriate section before selecting a response.

Materials & experimental systems			Methods		
n/a	Involved in the study		n/a	Involved in the study	
[ ]	[x]	Antibodies	[x]	[ ]	ChIP-seq
[ ]	[x]	Eukaryotic cell lines	[x]	[ ]	Flow cytometry
[x]	[ ]	Palaeontology and archaeology	[x]	[ ]	MRI-based neuroimaging
[ ]	[x]	Animals and other organisms			
[x]	[ ]	Human research participants			
[ ]	[x]	Clinical data			
[x]	[ ]	Dual use research of concern			

Antibodies

Antibodies used

p-MLC2 Cell Signaling Technologies #3671S, AB_330248
HIF-1alpha Cell Signaling Technologies #14179, AB_2622225
HIF-1alpha (ChIP grade) Cell Signaling Technologies #3716, AB_2116962
HIF-2alpha Cell Signaling Technologies #59973, AB_2799579
Normal Rabbit IgG Cell Signaling Technologies #2729, AB_1031062
CD-31 Cell Signaling Technologies #77699, AB_2722705
Vinculin Santa Cruz Sc-73264, AB_1131292
Inhibinalpha (R1) Biocare Medical SKU171
Anti-inhibin PO23 Oxford Brookes University
Anti-inhibin R1 Oxford Brookes University AB_2857371
TRC-105 Tracon Pharmaceuticals #754227
VE-cadherin (for IF) BD BioSciences #610252, AB_2276073
Goat [illegible]-globulin (NGG) Jackson ImmunoResearch #005-000-002, AB_2336984
Murine IgG anti-myc tag, 9E10 BioLegend #626802, AB_2148451
Chicken IgY anti-myc tag Merck Millipore #AB3252, AB_2235702
Rabbit IgG anti HA tag, HA.11 BioLegend #902302, AB_2565019
Murine IgG anti-HA tag, 12CA5 Roche Diagnostics #11666606001, AB_514506
Alexa 488-goat IgG anti rabbit IgG Invitrogen-Molecular Probes #R37116, AB_2556544
Alexa 546-goat (Fab')2 anti mouse IgG Invitrogen-Molecular Probes #A-11018, AB_2534085
Alexa 488-goat (Fab')2 anti rabbit IgG Invitrogen-Molecular Probes #A-11070, AB_142134
FITC-donkey IgG anti chicken IgY Jackson ImmunoResearch #703-095-155, AB_2340356
Cy3-donkey (Fab')2 anti mouse IgG Jackson ImmunoResearch # 715-166-150, AB_2340816

Validation	All validations are on manufacturers websites and no new non validated antibodies were used.

Eukaryotic cell lines

Policy information about cell lines

Cell line source(s)	HMEC-1 ATCC CRL-3243<br>HEK293 ATCC CRL-1573<br>OV-90 ATCC CRL-11732<br>OVCAR-5 NCI-60 N/A<br>PA1 ATCC CRL-1572<br>SKOV3 NCI-60 N/A<br>OVCA420 Susan Murphy N/A<br>ID8ip2Luc Jill Slack-Davis N/A<br>HEY Susan Murphy N/A<br>MEEC WT/ENG -/- Michelle Letarte N/A<br>COS7 ATCC CRL-1651
Authentication	Cell line authentication was performed at the Heflin Center for Genomic Science Core Laboratories at UAB by STR profiling
Mycoplasma contamination	Cells were routinely tested for mycoplasma contamination and used with 3-5 weeks of testing depending on the cell line
Commonly misidentified lines (See ICLAC register)	no commonly misidentified cell lines were used

Animals and other organisms

Policy information about studies involving animals; ARRIVE guidelines recommended for reporting animal research

Laboratory animals	Ncr nude female mice were used
Wild animals	Study did not involve wild animals
Field-collected samples	study did not involve samples collected from the field
Ethics oversight	UAB IACUC

Note that full information on the approval of the study protocol must also be provided in the manuscript.

Clinical data

Policy information about clinical studies

All manuscripts should comply with the ICMJE guidelines for publication of clinical research and a completed CONSORT checklist must be included with all submissions.

Clinical trial registration	Clinical data was not used from a clinical trial
Study protocol	Clinical data was not used from a clinical trial
Data collection	Clinical data was not used from a clinical trial
Outcomes	Clinical data was not used from a clinical trial
